# PE Teacher and Classmate Support in Level of Physical Activity: The Role of Sex and BMI Status in Adolescents from Kosovo

**DOI:** 10.1155/2015/290349

**Published:** 2015-08-25

**Authors:** Michal Bronikowski, Malgorzata Bronikowska, Ida Laudańska-Krzemińska, Adam Kantanista, Besnik Morina, Shemsedin Vehapi

**Affiliations:** ^1^Department of Didactics of Physical Activity, University School of Physical Education, Krolowej Jadwigi 27/38, 61871 Poznan, Poland; ^2^Department of Didactics of Physical Education, University of Pristina, Mother Therssa 1, 10000 Pristina, Kosovo

## Abstract

The aim of this paper is to examine the role of physical education (PE) teacher and classmate support in relation to sex and BMI status in adolescents' physical activity (PA) in Kosovo. A Classmate and Teacher Support Scale (with additional questions) was used on a cross-sectional sample of 608 girls and 620 boys aged 15–18, randomly selected from secondary schools of seven major municipalities in Kosovo. PA level was determined with a Physical Activity Screening Measure questionnaire. Descriptive statistics and a three-way ANOVA, along with Tukey's HSD post hoc test, were employed. The findings showed the levels of teacher and classmate support to be important factors in stimulating adolescents' PA. It was found that boys with normal weight, high support from teachers, and medium or high support from classmates were more physically active, compared with girls.

## 1. Introduction

The traumatic and devastating experiences of the Balkans conflicts disturbed the smooth development of all the nations on the region and the remnants of the war have influenced many spheres of life, including education. In an atmosphere of high ethnic tensions, as the one witnessed in Kosovo in the 1980s and 1990s, education was politicized: for Kosovar Albanians, a “parallel” education system was a crucial element in the process of maintaining and strengthening their separate and distinct national identity [1]. During the years of conflict Kosovar children were denied the right to learn in schools and instead relied on the separate “underground” education and health systems which were established [2]. In case of education this was carried out with limited (or severely damaged) infrastructure and the teaching of selected subjects only. In case of PE damage caused to already poor sporting infrastructure appeared to be one of the barriers of education. In schools, PE was introduced as an optional school subject firstly and became mandatory in 2006. In 2012 the subject was renamed “Health, Wellbeing and Physical Education” and has been reduced from 3 to 2 hours a week for all pupils in all grades, with one of these classes delivered in a sports gym and another in a classroom setting as a theoretical class [3]. In such circumstances the role of PE teachers and peers support turns to be of great importance in activating young generation. PA can be an important factor influencing the state of public health, specifically when considering recent generation of Kosovar adolescents brought up in the postwar era, and having limited access to sporting infrastructure and sports. Without understanding the potential associations in this area it is difficult to plan implementation of various health-related interventions.

Differences in cultural norms or potential differences in role of males and females in social life of such unique places (the Balkan nations), combined with time (postwar, postconflict), require the use of social ecological perspective. Therefore the transcontextual model [4], which hypothesizes that perceived autonomy support in PE would be transferred into autonomous motives in noneducational, after school settings, has been used as a theoretical framework for the present research. This model has been grounded in self-determination theory [4], which suggests that social factors support self-determination motivation and associated positive outcomes when psychological needs for competence, autonomy, and relatedness are met.

During adolescence, culture and society require adjustments in all of the aspects of daily living, including school, health care, social life, physical and mental development, and psychological well-being [5]. PA can have a potentially strong effect on children's and adolescents' behaviors [6, 7]. Rutten et al. [8] found that not only PE teachers, but also the physical school environment, plays a crucial role in promoting autonomous motivation by satisfying the pupils' need for autonomy. One of the major tasks of PE is to create a stimulating environment for learning skills and knowledge, enabling individuals to be physically active and healthy along the lifespan. Finding an optimal level of factors influencing this process is a key role of PE in schools [9–11]. PE has also been shown to be an important mediator of PA outside school [12, 13].

Findings suggest that peers and significant adults (parents, carers, and teachers) may take an important role in influencing youth PA [14]. Research also supports the associations between more positive PE experiences and higher levels of leisure-time PA in adolescents [15, 16]. Additionally, positive relationships with both teachers and peers are linked to optimal PE experiences [17]. Zhang et al. [18] found social support from friends, parents, and PE teachers as significant predictor of self-reported engagement in PA. Specifically, support from friends was found to be the most important social environmental factor. This makes sense from a developmental perspective as peer support in young adolescence is generally a dominant social factor and plays a critical role in the development of PA motivation and engagement and as such is also worth investigating in the context of Kosovar youth.

In a study on the postwar needs of children of Kosovo [19] it was found that only 12% of young adolescent girls, as compared to 39% of boys, declared participation in daily PA, whereas 20% of boys and 42% of girls declared none or rare participation in PA. Low participation in PA concerns girls, who are traditionally underrepresented in sport. This is due to the cultural tradition of Kosovo patriarchal familial relations remaining the basis of Kosovo's social functioning and solidarity [20]. To help overcome PA barriers teachers should create a positive and supportive environment in PE setting. This applies specifically to those with weight problems and less active girls. Recent research by Gjaka et al. [21] indicated that overweight and obesity in young children may not be a major concern threatening the revival of the educational or public health of Kosovo at present. According to Gjaka et al. [21] among 15-year-old teenagers 8% of girls and 10% of boys were categorized as overweight, with only 1-2% as obese. However, findings from a longitudinal study on a Swedish 8–18 years old population showed a sharp decline in average weight for height during war conflict and a rapid subsequent rebound thereafter [22].

It is well-documented that adolescents can gain numerous benefits from engaging in PA. Prochaska et al. [23] found parental and peer influences to be significant correlates of self-reported PA among middle school students, with peer support to be the strongest correlate in a regression model. Peer and family support were reported as significant predictors of involvement in PA in a study on a British cohort of students [24]. Social support has also been recognized for its influencing role in motivating youth [25], but there have been no studies on support and PA in Kosovo.

Thus, following the findings from other research, we designed a study to investigate the relationships of classmate and teacher support during PE lessons on moderate to vigorous physical activity (MVPA) of 15–18-year-old girls and boys, also taking into account the potentially mediating role of sex and the BMI status of students from Kosovo. This particular age group was chosen because research [11, 12, 25] shows that PA declines with age, with the steepest decline occurring between the ages 13 and 18 years. We targeted only secondary school students as the majority of secondary school students in Kosovar system of education fall within the ages 15–18 years. We also assumed they would be more reasonable and fair on their judgments on the social inner-group and with teacher relationships during PE classes.

## 2. Methods

### 2.1. Participants

The cross-sectional sample for the study included data from 608 girls between 15 and 18 years (M = 16.3, SD = 1.67) and 620 boys between 15 and 18 years (M = 16.7, SD = 1.83). The participants were recruited from randomly selected secondary schools in the seven major municipalities of Kosovo-Pristina (120 boys, 120 girls), Mitrovica (110 boys, 110 girls), Peja (90 boys, 90 girls), Gjakova (50 boys, 50 girls), Prizren (100 boys, 88 girls), Gjilan (100 boys, 100 girls), and Ferizaj (50 boys, 50 girls). The sample of students was selected through proportional stratified sampling. The sample unit was three randomly selected schools in each of the municipalities. The students were recruited at the schools' convenience from lessons where there was no specific syllabus content to be fulfilled. One intact class for each level was selected in each school to minimize potential disruption to the school curricular. Questionnaire administration was completed in one section that took approximately 20 minutes to complete, in whole class groups during one regular school hour in quiet classroom conditions. Students were also informed about the anonymous and voluntary nature of their participation.

### 2.2. Classmates and Teacher Support Measure

In the case of external support, two scales containing five questions each were designed to assess classmate and teacher support during PE lessons. These scales were based upon the Classmate and Teacher Support Scale, and the test-retest correlations were 0.69 [26, 27].

In our study we decided to adjust the original Teacher and Classmate Support Scale to the PE environment. The internal consistency of the scales was established using Cronbach's Alpha test. For the Teacher Support Scale it was *α* = 0.91, and for the Classmate Support Scale it was *α* = 0.87. Finally, the statements on the Classmate Support Scale were as follows: (1) other students accept me as I am, (2) most of the students in my class are kind and helpful, (3) the students in my class enjoy being together, (4) I get positive feedback from my peers when I play, and (5) I am often picked to play on various teams. The statements 1, 2, and 3 came from the original questionnaire [26]; the other statements were designed by the researchers. The items on the Teacher Support Scale were as follows: (1) our teacher treats us fairly; (2) when I need extra help, I can get it; (3) I get positive feedback from my teacher when I play; (4) our teacher makes sure we all treat one another fairly; and (5) the teacher lets us express our opinions. The statements 1 and 2 came from the original questionnaire [26]; the other three were designed by the researchers. A panel of international experts working in the EU project EuropeAid/130886/C/SER/KOS evaluated both instruments and translated the English version of the instruments into Albanian.

The examined individuals had to assess, on a 5-point Likert scale, whether they agreed (strongly agree) or disagreed (strongly disagree). The total score could amount up to 25 points on each of the scales. The categorization of support from teachers and classmates was made with the use of individual scores normalized to a sten scale [28], where individuals with 1–4 sten scores were classified as exhibiting a low level of support, those with scores of 5-6 as having a medium level of support, and individuals with 7–10 sten scores as receiving high support (Table 1).

### 2.3. Physical Activity Measure

The level of PA was determined using the Physical Activity Screening Measure [29]. This measure corresponds to the average number of days per week with at least 60 minutes spent partaking in various forms of PA during which, in the participants' subjective opinion, their heart rates increased, and they experienced a feeling of shortness of breath (higher breathing frequency). The scores ranged from 0 to 7 (days/week). Students were asked to answer two questions: (Q1) over the past 7 days, on how many days were you physically active for a total of at least 60 minutes per day? (Q2) Over a typical or usual week, how many days are you physically active for a total of at least 60 minutes per day? The MVPA index was calculated based on the following formula: MVPA = (Q1 + Q2)/2, where MVPA = PA index; Q1 = number of physically active days during the past 7 days; and Q2 = number of physically active days during typical (usual) week.

### 2.4. Height and Weight Measures

Body height and weight were self-reported by all participants and their body mass indexes (BMI) were calculated. BMI does not distinguish overweight due to excess fat mass from overweight due to excess lean mass and does not measure body fat directly. However, BMI does correlate to direct measures of body fat, such as underwater weighing and dual energy X-ray absorptiometry [30]. To be meaningful in children and adolescents, BMI measurements must be compared to a reference-standard that accounts for children's and adolescents' age and sex. Based on their BMI values, the participants were assigned to the following categories: (a) underweight, (b) normal weight, and (c) overweight, including individuals recognized as obese. Classification into the categories was based on age- and sex-adjusted cutoff values of BMI for children and adolescents as determined by Cole et al. [31, 32]. The final number of participants, stratified by sex and the frequency of underweight, normal weight, and overweight, is presented in Table 2.

### 2.5. Statistical Methods

It was examined whether the level of support from PE teachers, support from classmates, and various weight statuses were associated with the level of MVPA among girls and boys. To compare the individuals' MVPA level with the different levels of support they received (low, medium, and high), with different levels of body weight (underweight, normal weight, and overweight) in groups of girls and boys, a three-way (support × weight × sex) ANOVA was used. To conduct detailed multiple comparisons, Tukey's HSD post hoc test was employed. To determine the effect size for particular effects eta-squared was calculated. Statistical analysis was carried out using Statistica 10.0 software.

## 3. Results

Table 3 shows the MVPA level by teacher's and classmates' support, body weight status, and sex. It transpired that the main effect of teachers' support in PE on students' MVPA was significant (*F* = 26.32; df = 2; df = 1225; *P* < 0.001). Low support from PE teachers resulted in the low MVPA of students, whereas adolescents with medium (*P* < 0.001) and high support (*P* < 0.001) reported a higher level of MVPA. About 4% of variance on the MVPA level can be accounted for by this effect.

Classmate support in PE also had a significant effect (*F* = 51.75; df = 2; df = 1225; *P* < 0.001) on MPVA. The students receiving high support from classmates were more physically active, as compared with those receiving medium (*P* < 0.001) and low support (*P* < 0.001). About 8% of variance in MVPA level can be ascribed to this effect.

Furthermore, there was a statistically significant effect of body weight on MVPA (*F* = 4.07; df = 2; df = 1225; *P* < 0.05). Students in the normal body weight range were more physically active, compared to those who were overweight (*P* < 0.05). This effect explained less than 1% of variance in MVPA level. Sex also had a significant influence on MPVA (*F* = 109.02; df = 1; df = 1226; *P* < 0.001). The MVPA of boys was greater than that of girls. This effect explained about 8% of variance in MVPA level.

The 3-way interaction effect (sex, body weight, and support from teachers in PE) proved to have no statistically significant role (*F* = 1.40; df = 4; df = 1210; *P* = 0.234). The 2-way interaction effect of sex and support from PE teachers on MVPA was significant (*F* = 4.52; df = 2; df = 1222; *P* < 0.05). Only about 0.7% of MPVA level change resulted from this effect. Girls who received low support from teachers in PE manifested a lower level of MVPA than girls receiving high support. This was also the case for boys with high and medium support (*P* < 0.001) and for boys with low support (*P* < 0.02). Boys who received medium or high support from teachers in PE had a higher level of MVPA than their peers of both sexes who received low support (*P* < 0.000) (Table 4).

No statistically significant effect of 3-way interaction (sex, body weight, and classmates support in PE) on MVPA was discovered (*F* = 0.78; df = 3; df = 1211; *P* = 0.503). Similarly, no statistically significant role of the 2-way interaction effect of sex and classmates support on MVPA was observed (*F* = 1.89; df = 2; df = 1222; *P* = 0.150) (Table 5).

Teacher and classmate support in PE seem to be an important factor for the level of MVPA for both girls and boys in Kosovo. The 3-way interaction effect (support from teachers in PE and support from classmates in PE and sex) proved to have a statistically significant role (*F* = 2.71; df = 4; df = 1210; *P* = 0.029). Only about 1% of MPVA level change resulted from this effect. Boys who received high classmate support and high or medium teacher support were generally more physically active than other classmates (*P* < 0.05). Similarly, girls who received high classmate and high teacher support were more physically active than girls who received low or medium classmate support and low or medium teacher support (*P* < 0.05).

The 3-way interaction effect (support from teachers in PE, support from classmates in PE, and BMI status) also proved to have a statistically significant role (*F* = 2.67; df = 7; df = 1212; *P* = 0.010). About 2% of MPVA level change results from this effect. It was noticed that students with normal weight, high support from teachers, and medium or high support from classmates were more physically active (*P* < 0.05). Overweight students with low or medium support from teachers and classmates were less physically active (*P* < 0.05).

## 4. Discussion

Adolescents from both Europe and indeed around the world do not undertake PA at recommended levels [33]. This results in many attempts to improve PA in different settings and contexts. School is one of the settings where interventions aimed at PA enhancement and analyses of determinants of PA should be continuously studied. Girls require more specific attention because they are less physically active than boys. This is borne out in the results of the present study and many other investigations [34]. Goran et al. [35], also Sallis et al. [11], observed a decline in PA in teenagers, especially in girls before puberty, which was connected with earlier maturation of girls than boys. In the case of girls it was also associated with possible behavioral and environmental changes accompanying puberty, such as decreases in the accessibility of structured activity and in social desirability of PA, leading to a reduction in activity-related energy expenditure. Bélanger et al. [25] found that the maintenance of PA in adolescents is associated with supportive social environments, whereas a decline is associated with negative social validation, poor social support, and barriers related to access. Moreover, negative experiences were often reported to occur in the context of performance-based PA, which is often overly competitive and which is very prevalent in traditional methods of teaching of PE in Kosovo.

It is known that an increase in support from PE teachers and peers can increase motivation and the intention of students to undertake PA in their leisure time [36–38]. Based on an extended transcontextual model findings, the study of Hagger et al. [38] indicates a motivational sequence in which perceived autonomy support from teachers in a PE context and from peers and parents in a leisure-time PA context predicts autonomous motivation, intentions, and PA behavior in a leisure-time context. Specifically, adolescents who perceive teachers to support autonomous motivation in PE are more likely to report high levels of autonomous motivation in that context [38]. This needs to be taken into consideration with regard to the specific social and educational situation of Kosovo in which much depends on the school. Findings of a study on Kosovar school [39] indicate there is weak, unsatisfactory family-school interaction. Interestingly, if the success of students is supported by the school, the level of contact between family and the school is higher and the level of violence in school pupils is reported to be lower. This applies to primary school children and young teenagers; whereas with adolescents, school teachers and peers remain the most powerful source of influence, which was one of the reasons for choosing this age group of students in our study.

In the study presented here, a high level of both teacher and classmate support during PE classes was associated with higher levels of MVPA. However, this effect explained only 4% and 8% (resp.) of variance in the MVPA level. These results are consistent with the results of the study on Polish youth [40], which indicated that higher levels of classmate and teacher support in PE lessons were associated with higher levels of MVPA (explained only by maximum 2% of variance). As such, another source, or sources, of support may probably influence adolescents' PA. Zhang et al. [18] observed that significant predictors of PA were, in descending order of importance, friend support, parental support, and PE teacher support. Given that most PA behaviors occur in social and physical settings, Zhang et al. [18] stressed the importance of individuals' interaction with the physical and sociocultural environment, which are likely to influence an individual's choice to be physically active.

In our study, the interactional effect between sex, body weight, and classmate support in relation to MVPA was not statistically significant. This finding is inconsistent with the results of a study by Mikolajczyk and Richter [41] which indicates the importance of classmate support, which had a significantly stronger role in underweight, rather than overweight German youth, in relation to their PA. Kantanista et al. [40] have also indicated that high levels of teacher and classmate support were associated with higher MVPA among underweight (only in the case of classmate support, normal weight girls, and underweight and normal weight boys). Interestingly, the level of MVPA among Kosovar youth in both overweight girls and boys was not differentiated by the level of teacher and classmate support in PE. The highest level of MVPA was found in boys with normal weight receiving high or medium support from the teachers, whereas the lowest level was recorded in girls with low support, regardless of body weight status. A similar pattern was found in the case of classmate support.

Fostering and stimulating social awareness on PA, or indeed health in a public sense, can serve many purposes in the development of regions suffering from national or ethnic disturbances. Calhoun [42] states that in case of the Balkans “ethnic or national solidarities may be created not only with the purpose of engaging groups in conflicts as a matter of exclusion by the powerful, but, as well, they may serve as a resource for collective and reciprocal support among the less powerful and disadvantaged groups.” People of Kosovo have proven they are open to new challenges in public health interest when the Ministry for Culture, Youth and Sport started organizing Priŝtina's Half-Marathon. Since 2000, the number of participants has grown to 1400 (in 2014) and has helped to promote peaceful coexistence in the region by encouraging participation of all individuals despite national, ethnic, or religious origins, which may result in long-term health outcomes. Another initiative called Football 4 peace, run by the Sports Sans Frontiers Foundation and aided by the Ministry of Culture, Youth and Sport, aimed at bringing together children from different ethnic and religious backgrounds around Kosovo. Likewise, in the educational sphere, foundations like “Atmosfera” (started in Kosovo in 2004) try to open up space for religious and cultural dialogue and reconciliation. The activities of the foundation are aimed at engaging Kosovar youth in educational and cultural activities to help them preserve their own culture, history, and religion based on an ethos of mutual respect [43]. It shows that support may play not just a motivating, but also a socializing role, which is so much needed by this generation of Kosovar young adolescents that lacks the proper socialization that usually occurs through a well-organized educational system. There needs to be a change in educational practices from the competitive, performance-related traditional model [3] towards life-style and health-oriented one. This would be more likely to lead to higher levels of engagement of Kosovar youth, both boys and girls, in those activities and skills that are essential for life-long PA participation and as such would enhance public health in general aspects.

The strengths of this study include a contribution to the literature on social support and PA, especially with regards to the aspects of supporting underweight, normal body weight, and overweight adolescents in Kosovo. Reliance on self-reporting methods to measure social support and PA is a potential limitation and can be subject to response bias. Furthermore, self-reported body weight and height may be also affected by recall bias. As reported in many previous studies, underestimation of weight and overestimation of height may lead to subsequent underestimation of BMI [44, 45]. Also we need to acknowledge some potential limitations associated with translating the questionnaire in Albanian, though it has been done by a group of experienced international experts from both outside and inside Kosovo. However, it has to be noted that based on results from 11–16-year-old girls and boys from seven different countries Torsheim et al. [27] indicated that the Teacher and Classmate Support Scale is well suited for use in large social surveys and can be used in spite of potential language and cultural differences.

## 5. Conclusions

According to our knowledge it is the first study on social support and PA in Kosovo. The context of our study, incorporating PE lessons in Kosovo, should likewise be considered as unique. It is clear that in order to enhance and positively influence middle school students' participation in MVPA, a supportive social context is required, especially in the case of Kosovar girls, who, due to cultural and traditional roles in this society, need more attention and support. However, in order to understand the positive predictive strengths of factors influencing leisure-time PA of Kosovar adolescents, a specific social and physical environmental perspective needs to be applied.

## Figures and Tables

**Table 1:** Number of girls and boys, stratified by the occurrence of low, medium, and high levels of support from teachers and classmates.

Sex	Low support	Medium support	High support	Total participants
	*n* (%)	*n* (%)	*n* (%)	*n* (%)
Girls				
Support from teachers	178 (29.3)	252 (41.4)	178 (29.3)	608 (100)
Support from classmates	258 (42.4)	226 (37.2)	124 (20.4)	608 (100)
Boys				
Support from teachers	136 (21.9)	270 (43.6)	214 (34.5)	620 (100)
Support from classmates	162 (26.1)	210 (33.9)	248 (40.0)	620 (100)
Total				
Support from teachers	314 (25.6)	522 (42.5)	392 (31.9)	1228 (100)
Support from classmates	420 (34.2)	436 (35.5)	372 (30.3)	1228 (100)

**Table 2:** Number of girls and boys, stratified by the occurrence of underweight, normal weight, and overweight.

Sex	Underweight	Normal weight	Overweight	Total
	*n* (%)	*n* (%)	*n* (%)	*n* (%)
Girls	58 (9.5)	516 (84.9)	34 (5.6)	608 (49.5)
Boys	28 (4.5)	498 (80.3)	94 (15.2)	620 (50.5)
Total	**86 (7.0)**	**1014 (82.6)**	**128 (10.4)**	**1228 (100)**

**Table 3:** Descriptive statistics of MVPA for students with different levels of support from teachers and classmates in PE and their different weight status and sex (M: mean, SD: standard deviation, and SEM: standard error of the mean).

Factor	Factor level	*N*	MVPA (days/week)		
			M	SD	SEM
Teacher support	Low	314	1.95	0.95	0.05
	Medium	522	2.48	1.28	0.06
	High	392	2.58	1.35	0.07

Classmate support	Low	420	2.13	1.02	0.05
	Medium	436	2.17	1.07	0.05
	High	372	2.91	1.51	0.08

Weight status	Underweight	86	2.41	1.12	0.12
	Normal weight	1014	2.41	1.25	0.04
	Overweight	128	2.08	1.31	0.12

Sex	Girls	608	2.02	0.90	0.04
	Boys	620	2.73	1.43	0.06

**Table 4:** Descriptive statistics of MVPA for girls and boys with different levels of support from PE teachers (M: mean, SD: standard deviation, and SEM: standard error of the mean).

Sex	Teacher support	*N*	MVPA (days/week)		
			M	SD	SEM
Girls	Low	178	1.76	0.92	0.07
	Medium	252	2.01	0.79	0.05
	High	178	2.27	0.98	0.07

Boys	Low	136	2.19	0.94	0.08
	Medium	270	2.92	1.48	0.09
	High	214	2.84	1.55	0.11

**Table 5:** Descriptive statistics of MVPA for girls and boys with different levels of support from classmates (M: mean, SD: standard deviation, and SEM: standard error of the mean).

Sex	Classmates support	*N*	MVPA (days/week)		
			M	SD	SEM
Girls	Low	258	1.87	0.87	0.05
	Medium	226	1.96	0.76	0.05
	High	124	2.42	1.09	0.10

Boys	Low	162	2.54	1.09	0.09
	Medium	210	2.39	1.28	0.09
	High	248	3.15	1.64	0.10
